# The y-ome defines the 35% of *Escherichia coli* genes that lack experimental evidence of function

**DOI:** 10.1093/nar/gkz030

**Published:** 2019-01-30

**Authors:** Sankha Ghatak, Zachary A King, Anand Sastry, Bernhard O Palsson

**Affiliations:** 1 Bioengineering Department, University of California, San Diego, La Jolla, CA 92093, USA; 2 Department of Pediatrics, University of California, San Diego, La Jolla, CA 92093, USA; 3 Novo Nordisk Foundation Center for Biosustainability, Technical University of Denmark, Kemitorvet, Building 220, 2800 Kongens, Lyngby, Denmark

## Abstract

Experimental studies of *Escherichia coli* K-12 MG1655 often implicate poorly annotated genes in cellular phenotypes. However, we lack a systematic understanding of these genes. How many are there? What information is available for them? And what features do they share that could explain the gap in our understanding? Efforts to build predictive, whole-cell models of *E. coli* inevitably face this knowledge gap. We approached these questions systematically by assembling annotations from the knowledge bases EcoCyc, EcoGene, UniProt and RegulonDB. We identified the genes that lack experimental evidence of function (the ‘y-ome’) which include 1600 of 4623 unique genes (34.6%), of which 111 have absolutely no evidence of function. An additional 220 genes (4.7%) are pseudogenes or phantom genes. y-ome genes tend to have lower expression levels and are enriched in the termination region of the *E. coli* chromosome. Where evidence is available for y-ome genes, it most often points to them being membrane proteins and transporters. We resolve the misconception that a gene in *E. coli* whose primary name starts with ‘y’ is unannotated, and we discuss the value of the y-ome for systematic improvement of *E. coli* knowledge bases and its extension to other organisms.

## INTRODUCTION

Unannotated genes in model organisms still play important roles in determining cell phenotype. This point was driven home by recent efforts to synthesize a minimal bacterial genome. The resulting syn3.0 organism includes just 473 genes, all of which are essential for growth, and a full 30% of which lack functional annotation (1,2). Even in *Escherichia coli* K-12 MG1655, perhaps the best-studied model organism, unannotated genes often appear in experimental studies of strain engineering (3), laboratory evolution (4) and pathogenicity (5). Efforts to build predictive models of the genotype-phenotype relationship for whole cells will be hindered by unannotated genes that still affect cell phenotype (6,7).

Historically, unannotated genes in *E. coli* are known as ‘y-genes’ because they have primary names starting with ‘y’ (8)—not to be confused with ‘Y genes’ which can indicate genes on the human Y chromosome (9). However, genes with primary names that begin with ‘y’ are often functionally annotated. For example, in a recent study where *E. coli* was engineered to produce fatty acids via reversal of the fatty-acid beta-oxidation pathway, the authors knocked out the genes *yqeF* and *yqhD* to increase production of target molecules (3) and included the genes *ydiQRST, ydiO* and *ydbK* in a predictive model of the cell (10). Searching for these genes in public knowledge bases such as EcoCyc (11) reveals that they vary greatly in annotation quality. Some (e.g. *yqhD*) are well-annotated with direct experimental evidence, while others (e.g. *ydiO*) have limited functional information. The variation of annotation quality among y-genes suggests that a systematic approach to understand the unannotated genes in *E. coli* is needed which goes beyond the primary gene name.

There are several knowledge bases that represent the collected knowledge of the *E. coli* K-12 MG1655 genome: EcoCyc (11), EcoGene (12), UniProt (13) and RefSeq (14). Other useful knowledge bases cater to specific classes of gene products, such as the RegulonDB, which contains manually curated functional information about transcription factors in *E. coli* (15). Our initial review of these knowledge bases yielded conflicting information on gene function and level of annotation for many *E. coli* genes. Any attempt to systematically assess the function of unannotated genes must therefore draw from multiple knowledge bases and resolve these conflicts.

Many research groups have categorized *E. coli* genes and proteins by annotation quality as a part of their studies. In 2009, Hu *et al.* constructed a global functional atlas of *E. coli* proteins (16). First, they identified all unannotated proteins in the K-12 W3110 and MG1655 genomes. In order for a protein-encoding gene to be considered functionally uncharacterized in their analysis, it had to meet the following criteria: (i) The gene name begins with ‘y’, (ii) the gene does not have a known pathway within EcoCyc and (iii) the gene does not have a functional description in GenProtEC (17) (any gene with a description containing the words ‘predicted’, ‘hypothetical’, or ‘conserved’). Based on these criteria, it was determined that 1431 of 4225 protein coding sequences were not functionally annotated. In 2015, Kim *et al.* published a database called EcoliNet that curated and predicted cofunctional gene networks for every protein coding gene in the *E. coli* genome (18). This study also quantified the number of uncharacterized protein coding genes in *E. coli*. To assess functional annotation, they used the presence of experimentally supported ‘biological process’ annotations in the Gene Ontology database (19). They concluded that ∼2000 protein coding genes in *E. coli* were not functionally annotated. The most comprehensive effort to assess the level of annotation in bacterial genomes has been Computational Bridges to Experiments (COMBREX) (20,21). The COMBREX knowledge base currently contains information about 4182 protein coding genes in *E. coli* K-12 MG1655, of which 2378 (57%) have experimentally verified function, 1741 (42%) have predicted but not experimentally verified function and 63 (2%) have no predicted function. These studies of unannotated genes in *E. coli* K-12 MG1655 provided inspiration for this work. However, our effort covers both protein-coding and nonprotein-coding genes, disregards nomenclature (i.e. whether a gene name begins with ‘y’) as an indicator of annotation quality, and is presented as a reproducible workflow to keep the analysis up-to-date as knowledge bases improve.

In seeking the set of *E. coli* genes that lack functional annotation, one must determine what level of annotation is sufficient to call a gene ‘well-annotated.’ Experimental evidence is essential for assigning gene functions with confidence. While computational inference of gene function is improving, it is still prone to error (22,23), it cannot distinguish the various roles played generalist proteins in different environments (24,25), and it cannot be used to determine the detailed effects of genes like transcription factors with complex modes of action (26). Taking the example of transcription factors, computational inference *can* be used to predict that a gene is a transcription factor (26,27). Should this be sufficient to determine that the gene is annotated?

To answer this question, we propose that functional annotation of a gene should establish a mechanistic link to the phenotypic effects of the gene. Thus, a transcription factor is annotated if the regulated genes are known and the mode of regulation (activation, repression) for each gene is established. To take another example, knowing that a gene encodes an oxidoreductase enzyme does not indicate which phenotypes might be enabled by the gene (e.g. which catabolic pathway it might be a part of). But if a specific biochemical activity can be established (e.g. by enzymatic assay), that could clearly establish the contribution of the gene to cell phenotype. Furthermore, an association between a gene and a phenotype is not sufficient if it lacks the mechanistic information that is generally desired when annotating genes (e.g. if a gene is essential for cell division but the mechanism of the effect is unknown, then the gene fails this test). This approach at least provides guidelines on how to assess the level of functional annotation, even if it is difficult to put into practice. This article describes a first approximation of the approach, but to precisely define and enforce this definition of functional annotation will require continued effort, and we describe the next steps in the Discussion.

We propose the term ‘y-ome’ for the set of genes in a genome that lack experimental evidence of function with a mechanism for affecting cell phenotypic. We sought to identify the y-ome of *E. coli* K-12 MG1655 based on existing knowledge bases. Because we are limited by the annotations that are already available, this y-ome is an approximation based on a heuristic workflow described below. Therefore, we present the *E. coli* y-ome as a reproducible workflow that can improve over time.

The resulting y-ome includes 35% of *E. coli* genes. We describe some trends for these y-ome genes, including their enrichment in the termination region of the *E. coli* chromosome, lower average expression levels than well-annotated genes and evidence that certain types of genes (e.g. transporters) are enriched in the y-ome. Finally, we resolve the misconception that a gene in *E. coli* whose primary name starts with ‘y’ is necessarily unannotated.

## MATERIALS AND METHODS

### A heuristic approach to identifying the y-ome

A y-ome workflow was developed to assign genes to the y-ome based on annotations in *E. coli* knowledge bases. Existing knowledge bases do not explicitly annotate whether a gene has both experimental evidence of function and sufficient evidence to determine the mechanistic effect on cell phenotype. Therefore, we took a heuristic approach to analyze the databases, following the process that one might take if they were manually curating the entire list.

First, we looked for common indications in annotations that genes were very poorly annotated. Often particular keywords or structured annotations were identified—e.g. the keyword ‘hypothetical’ in a gene description—that always appeared along with genes that had minimal annotation. Next, we looked for clear indications that genes were very well annotated, such as entries in a functional evidence ontology. Defining these keywords and annotation rules was a subjective process, so we took a conservative approach whenever possible. Genes that could not be automatically annotated were labeled for manual curation. The full process is detailed in the following sections.

### A workflow to determine the *E. coli* y-ome

We collected data from the following knowledge bases: EcoCyc release 22.5 (11), EcoGene version 3.0 (12), UniProt release 2018_10 for proteome UP000000625 (13) and RegulonDB version 9.4 (15). While data in RegulonDB is also available in EcoCyc, the RegulonDB data downloads were more convenient for extracting annotations of transcription factors. The *E. coli* K-12 MG1655 NCBI RefSeq genome annotation (accession NC_000913.3) was included for comparison as it is a commonly-used resource in the field (14). Features were extracted from the downloaded data and used to populate a relational (SQLite) database.

Pseudogenes are genes that have lost their function through mutation, and phantom genes are regions of the genome that were once considered genes but are no longer based on better evidence or analysis. Pseudogenes and phantom genes are not included in the y-ome, so they are assigned to the ‘Excluded’ category in the workflow. Annotations of pseudogenes and phantom genes were taken from both EcoGene and EcoCyc. In EcoGene, pseudogenes are indicated by a primary name ending in an apostrophe. EcoCyc explicitly defines lists of pseudogenes and phantom genes, currently available here:

- https://ecocyc.org/ECOLI/class-instances?object=Pseudo-Genes
- https://ecocyc.org/ECOLI/class-instances?object=Phantom-Genes

Cryptic genes—defined as genes that are phenotypically silent—were not marked ‘Excluded’. These genes might be activated under novel conditions, and therefore they are an interesting component of the y-ome (28).

Keywords were used to automatically categorize genes for each knowledge base feature. To identify the keywords, we read gene entries in the knowledge bases to look for commonly used phrases in the parlance of the particular knowledge base that signified the level of annotation. For example, in EcoCyc, the keywords ‘possibly’, ‘predicted’ and ‘hypothetical’ in the ‘description’ field were used to identify genes with low annotation level, and the keywords ‘assay’, ‘traceable author statement to experimental support’ and ‘reaction blocked in mutant’ in the ‘evidence’ field were used to identify genes with high annotation (well-annotated). The full list of keywords used in the workflow listed in Dataset S5.

Defining these keywords was a subjective process, so we relied on structured data whenever possible, with the following rules. To determine annotation level for UniProt genes, we used the ‘annotation score’ for each associated protein. Annotation scores of two or below were used to indicate ‘y-ome’ and four or above to indicate ‘well-annotated’. Genes with annotation score three were categorized as ‘Not enough information for automated assignment’. Additionally, EcoCyc genes with a reaction_equation annotation, gene complex annotations or explicitly marked as insertion elements were considered ‘well-annotated’.

### Consensus rules

After assigning genes in each knowledge base to categories, consensus rules were applied to combine the results from the separate knowledge bases. In general, we checked first for agreement among knowledge bases. For instance, if two knowledge bases indicated a gene was ‘well-annotated’ and the others did not have enough information to assign a category, then the consensus was ‘well-annotated’. When databases disagreed, then no consensus was possible. In these cases, manual annotations were made (for 334 genes) based on reading the knowledge base entries and consulting the literature. There were four exceptions that were identified as heuristics to improve the quality of the final list:

1. The Evidence section for EcoCyc genes is a high-quality, manually-curated ontology of functional evidence derived from the primary literature (29). Therefore, we gave this section priority over other data in the workflow. Particularly, we looked for Evidence features with keywords ‘assay’, ‘reaction blocked in mutant’ and ‘traceable author statement to experimental support’ which were marked as Well-annotated in the final categorization.
2. RegulonDB contains curated and experimentally-validated annotations of transcription factors. Thus, genes with ‘Strong’ evidence in RegulonDB were marked as well-annotated in the final categorization.
3. When EcoCyc and UniProt were both categorized as well-annotated for a given gene, then this gene was automatically marked as well-annotated in the final categorization. This heuristic was helpful to identify cases where EcoGene was missing key evidence that the other knowledge bases had picked up (e.g. *dhaM*).
4. Insertion elements, identified in EcoCyc by gene names beginning with ‘ins’, were considered to be well-annotated in the final categorization.

### Genes with no information

To identify genes for which no information at all is available, we filtered the database for genes with features drawn from knowledge-base-specific phrase lists that corresponded to genes with no other functional information. For example, ‘Putative uncharacterized’ often appeared in such UniProt entries. As another example, EcoCyc genes with no information have summaries that begin with this phrase ‘No information about this’. (e.g. ‘No information about this protein was found by a literature search conducted on 23 February 2017’ for *ybiU*). The full list of phrases that were used can be found in Dataset S5.

When genes were annotated only with a protein domain or family, we still included them in the list because such domains (e.g. DUF1479 for *ybiU*) often themselves have no functional information associated (DUF1479 has the description ‘Protein of unknown function’ on Pfam: https://pfam.xfam.org/family/PF07350).

### Gene expression compendium

A compendium of RNA-seq data for *E. coli* K-12 MG1655 and BW25113 (wild-type, single gene mutants and laboratory evolution endpoints) was used to analyze expression of y-ome genes. All RNA-seq experiments were conducted using the protocol described by Seo *et al.* (30). Raw sequencing reads were collected from GEO (31) (see Dataset S4 for accession numbers) and mapped to the reference genome (NC_000913.3 for strain MG1655 and CP009273 for BW25113) using bowtie 1.1.2 (32) with the following options: ‘-X 1000 -n 2 -3 3’. Transcript abundance was quantified using *summarizeOverlaps* from the R GenomicAlignments package, with the following options: ‘mode = ‘IntersectionStrict’, singleEnd = FALSE, ignore.strand = FALSE, preprocess.reads = invertStrand’ (33). Transcripts per Million (TPM) were calculated by DESeq2 (34). The final expression compendium was log-transformed with log_2_(TPM+1) before analysis, referred to as log-TPM. Biological replicates with R^2^ < 0.9 between log-TPM were removed to reduce technical noise.

### Co-expression analysis

Gene co-expression analysis was performed on the gene expression compendium using IterativeWGCNA (35), an extension of the popular WGCNA algorithm (36). IterativeWGCNA uses iterative pruning steps to improve the robustness of detected gene modules. The method was run with a minimum module size of 10 and otherwise with default parameters.

### Chromosome location & density

Gene locations were extracted from gene start sites in the *E. coli* RefSeq genome annotation NC_000913.3. Gene density plots were created with a circular kernel density estimation method using the von Mises distribution.

## RESULTS

### A workflow for identifying the *E. coli* y-ome

**Figure 1.**
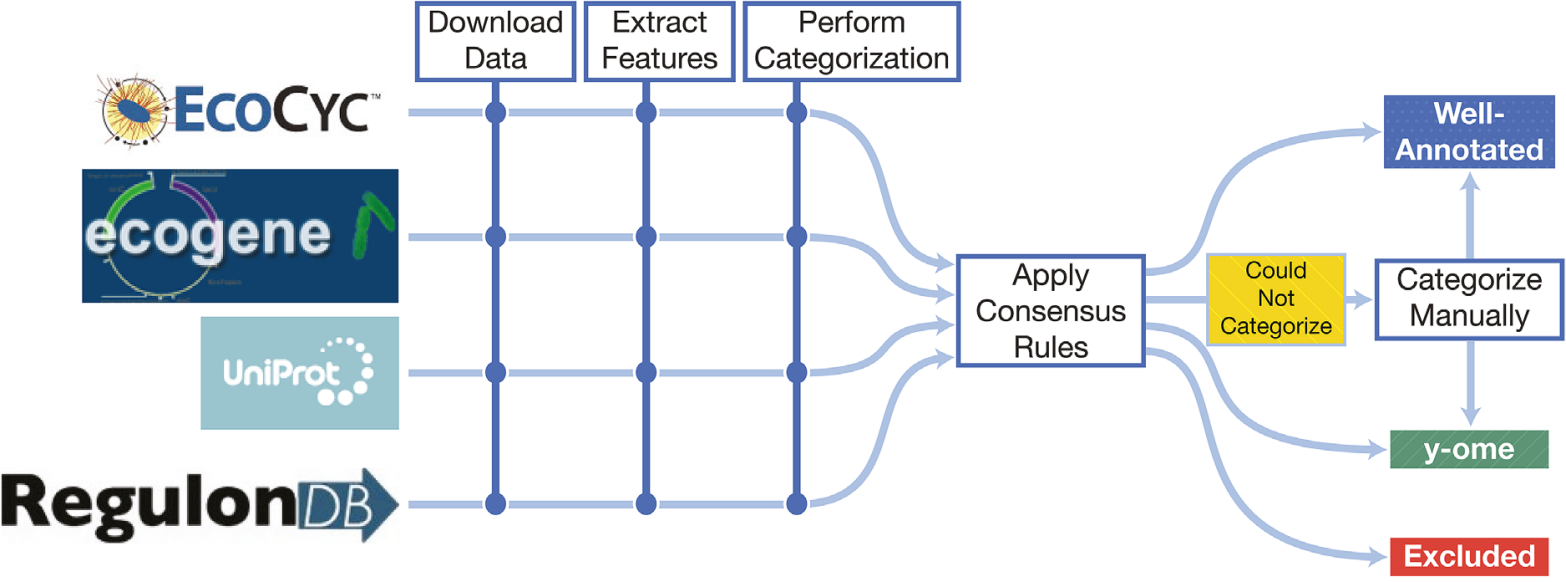
A workflow for defining the y-ome of *E. coli* K-12 MG1655. Data were collected from four *E. coli* knowledge bases, and automated categorization was applied to determine their annotation level. Next, consensus rules were applied to combine categorizations from multiple databases. When the consensus rules could not be applied, genes were manually curated and placed in one of the categories. Thus, genes were categorized as ‘Well-Annotated’ or ‘y-ome’ according to the definition of the y-ome (see Section ‘Definition of the y-ome’). Pseudogenes and phantom genes were treated separately in the ‘Excluded’ category.

**Figure 2.**
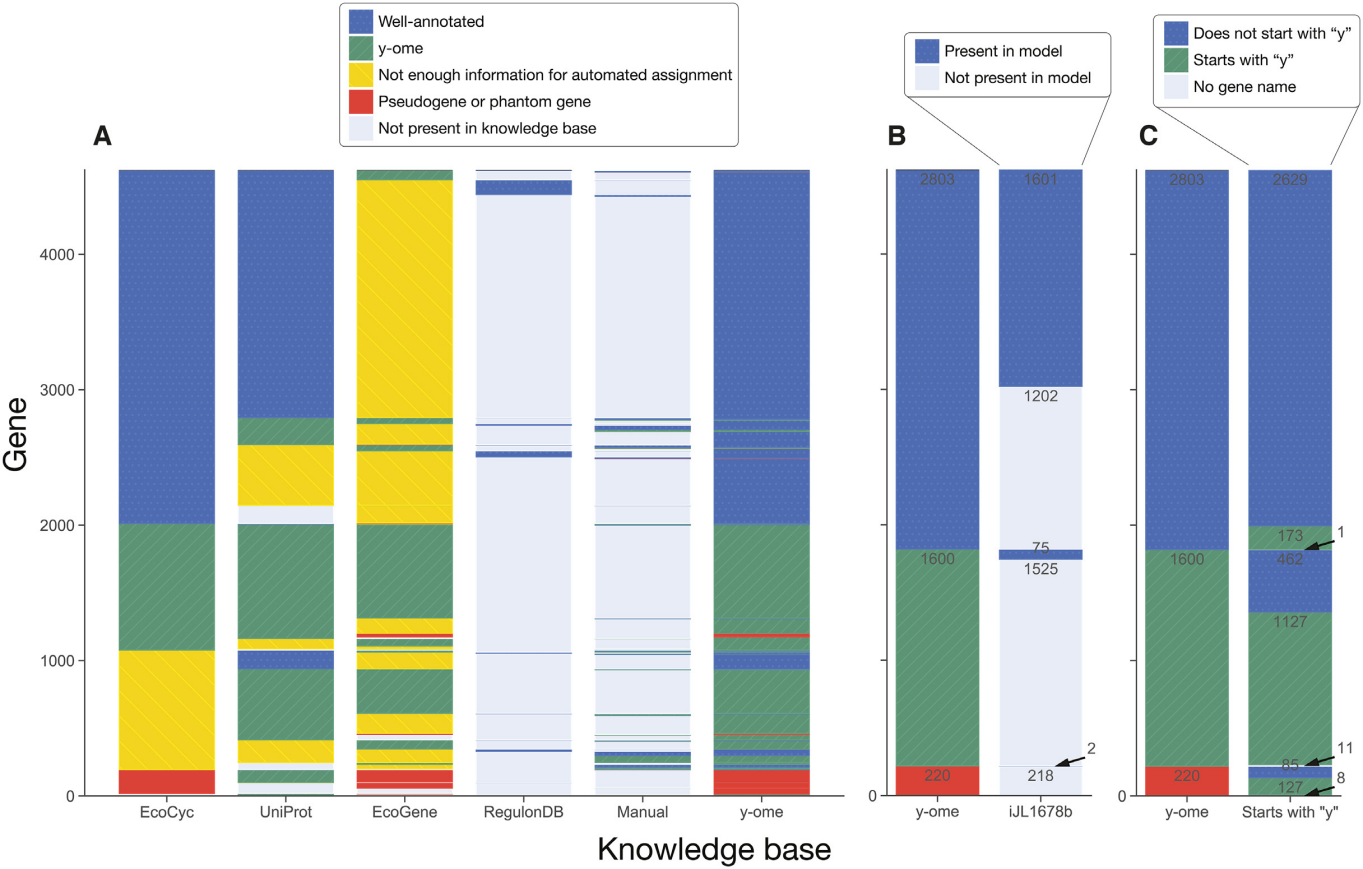
Gene annotation across knowledge bases. The y-axes represent all unique genes in the database. Gene order is maintained within each subplot so one can track the annotation of a set of genes across knowledge bases. (**A**) An automated approach was used to categorize genes from each database as ‘Well-annotated’ or ‘y-ome’ based on the definition of the y-ome. Pseudogenes and phantom genes were excluded. The resulting y-ome includes 1600 genes. (**B**) y-ome categories were compared to the content of the latest *E. coli* genome-scale ME-model. (**C**) A total of 173 genes have primary names that start with ‘y’ but are well-annotated, and 462 genes in the y-ome have non-‘y’ primary names.

To systematically determine an initial y-ome for *E. coli* K-12 MG1655, we developed a semi-automated approach (Figure 1) to identify unique genes across four *E. coli* knowledge bases and integrate their annotations. The automated part of this process proceeded in three steps: (i) downloading data from each knowledge base, (ii) extracting text-based features (Dataset S2) and (iii) using keywords to automatically assign each gene annotation in a knowledge base to the categories ‘y-ome,’ ‘Well-annotated’ or ‘Not enough information for automated assignment’ (Figure 2A). Pseudogenes and phantom genes were kept separate and marked ‘excluded’. The rules and keywords used to make these assignments are described in the ‘Materials and Methods’ section.

Based on this analysis, we identified 4623 unique genes across *E. coli* K-12 MG1655 knowledge bases, and each was assigned to the ‘y-ome’, ‘well-annotated’ or ‘excluded’ categories (Dataset S1). Of these 4623 genes, 2803 have information that indicate a sufficient level of functional evidence to exclude them from the y-ome (Figure 2A), and 1600 genes (34.6%) are in the y-ome of *E. coli* K-12 MG1655. No individual knowledge base provides information to fully define the y-ome, but EcoCyc comes the closest (Figure 2A). Of the 1600 y-ome genes, there were 111 for which we found no information in the knowledge bases (see ‘Materials and Methods’ section) and 220 that were marked as pseudogenes or phantom genes.

Of the similar studies reviewed in the Introduction, only the article by Hu *et al.* (16) provided a complete list of unannotated genes. Comparing that set to the y-ome reveals differences in the annotation levels of hundreds of genes, particularly in the cases where Hu *et al.* relied on the primary name (names ending in ‘y’) to determine the annotation level of the gene (Supplementary Figure S1). By this measure, at least, the y-ome workflow offers a more complete view of the annotation level of *E. coli* genes.

### Gene expression and chromosome location

**Figure 3.**
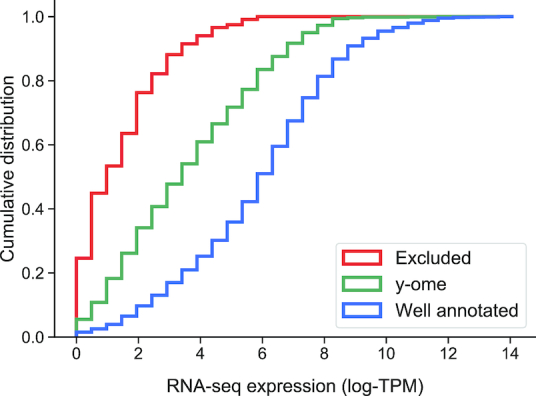
Average gene expression for all genes in a compendium of *E. coli* RNA-seq data. Cumulative distributions of normalized mean expression levels (mean log-TPM) for ‘y-ome’ (green), ‘Well annotated’ (blue) and ‘Excluded’ (red) genes across the 78 conditions surveyed in a compendium of RNA-Seq data.

It was previously observed by Hu *et al.* that poorly annotated genes tend to be expressed at a lower level than well-annotated genes (16). We confirmed this with the y-ome by comparing gene expression of y-ome genes and well-annotated genes in a compendium of RNA-seq data. The RNA-seq compendium includes expression values for 4385 *E. coli* genes across 78 conditions, including a variety of carbon sources, nitrogen sources, gene knockouts, stress conditions and laboratory evolution endpoints (conditions are described in Dataset S4). Genes in the y-ome tend to have lower expression across the surveyed conditions (Figure 3), where the *t*-test *P*-value < 1 × 10^−6^. A large-scale quantitative proteomics dataset is also available for *E. coli* (37), and comparing y-ome protein abundance to well-annotated protein abundance in that dataset reveals the same trend (Supplementary Figure S2). Attempts to annotate y-ome genes may be more successful if priority is given to the highly expressed y-ome genes that have a greater potential to affect observable phenotypes. Alternatively, experiments that identify growth conditions with greater expression of y-ome genes could help elucidate their functions because genes are more likely to have a phenotypic effect under conditions where they are expressed (28).

**Figure 4.**
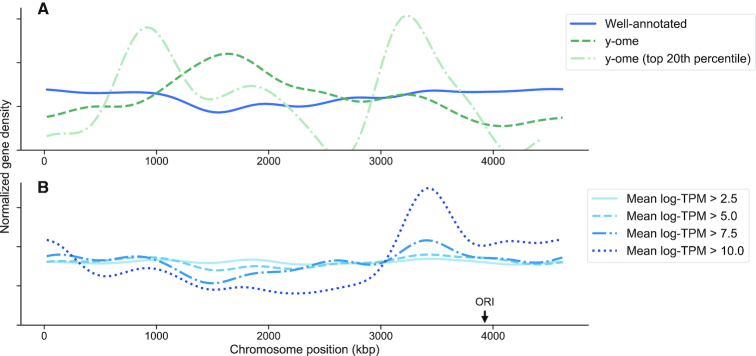
Gene expression by location on the chromosome. (**A**) The y-ome genes are enriched in the termination region of the *E. coli* chromosome, opposite the ORI. However, the y-ome genes in the top 20th percentile of expression (mean log-TPM > 7.57) are enriched both near the ORI and the termination region. (**B**) Highly expressed genes are known to be enriched around the ORI (16), which we confirmed by plotting density of genes in the chromosome with increasing thresholds of mean gene expression (mean log-TPM) across the compendium of RNA-seq data for 78 conditions.

We observed a low density of y-ome genes near the origin of replication (ORI) of the *E. coli* chromosome and a high density of y-ome genes in the termination region (opposite ORI, Figure 4A). Highly expressed genes are known to be enriched near ORI (38–40), which was observed in our gene expression compendium (Figure 4B). It has also been shown that genes whose deletion affect growth phenotypes under stress conditions, so-called ‘responsive genes,’ are enriched near ORI (41). These observations tell a simple story of highly expressed genes that have obvious effects on phenotypes under laboratory conditions and are therefore well-annotated, and lowly expressed genes that do not affect phenotypes enough to be easily characterized. However, the y-ome genes with highest mean expression (top 20th percentile) are split between the origin and termination regions (Figure 4A), which suggests that some other factor might be keeping genes near the termination region from being characterized. High-throughput gene annotation might shed further light on this phenomenon.

### Co-expression analysis

Genes that are co-expressed often have functional relationships, so weighted-gene correlation network analysis (WGCNA) is a popular approach for generating hypotheses of gene functions (18,36). Using an extension of the method called IterativeWGCNA (35), we identified co-expressed gene modules in the RNA-seq compendium.

**Figure 5.**
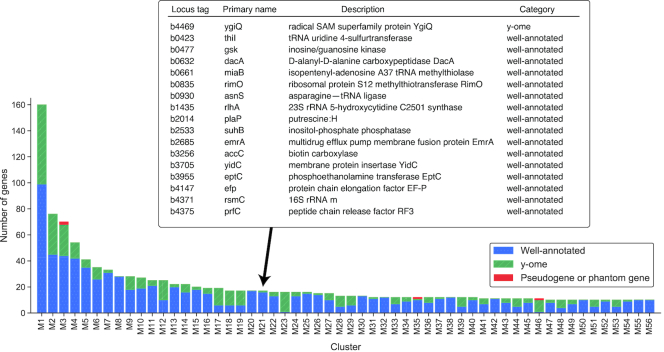
Co-expressed gene modules identified with IterativeWGCNA. The bar plot summarizes the number of genes in each module by category (‘Well-annotated’, ‘y-ome’ and ‘Psuedogene or phantom gene’). The table lists all genes in Module M21—of which only ygiQ is in the y-ome—and the primary names and descriptions from EcoCyc.

Well-annotated and y-ome genes tend to appear in modules together, indicating that highly-expressed y-ome genes could be functioning to affect cell phenotypes (Figure 5, Dataset S6). In total, 840 well-annotated genes and 347 y-ome genes appeared in the 56 modules (along with 4 genes marked as pseudogenes or phantom genes; these might actually have functional roles).

As an example, module M21 includes the y-ome gene *ygiQ* whose only annotation is membership in the radical SAM protein superfamily (Figure 5). All other genes in the module are well-annotated, and they are generally associated with protein translation (e.g. ribosomal subunits, protein elongation factors and transfer RNA-associated proteins). Some radical SAM enzymes are known to be involved in protein translation, e.g. *rlmN* (42). These observations provide some insight into the potential function of *ygiQ*. This study is not intended to determine new y-ome functions. Rather, the co-expression analysis demonstrates that y-ome genes are often expressed with well-annotated genes, and these 347 co-expressed y-ome genes are the highest priority targets for experimental characterization (Dataset S6).

### Functions of y-ome genes

The most common terms associated with y-ome genes can easily be extracted from *E. coli* knowledge bases (Table 1). These terms indicate that many membrane-associated proteins (502 genes) and particularly transporters (295 genes) remain to be annotated. Membrane-bound proteins and transporters are particularly hard to characterize with certainty (43), but high-throughput methods might change that, as they already have for enzymatic assays (44,45), gene-environment networks (46) and protein–protein interactions (16). Thus, the y-ome offers a set of candidate transport-associated genes for high-throughput analysis. High-throughput analysis could also be relevant for gene sets related to enzymes (271 genes), signaling (267 genes), lipoproteins (98 genes) and biofilms (74 genes). As evidence accumulates in *E. coli* knowledge bases, this workflow can be run again to improve the candidate gene sets.

**Table 1.** The most common words found in knowledge bases features for y-ome genes.

Word or word set	y-ome (*n* = 1600)	Well-annotated (*n* = 2803)	Excluded (*n* = 220)
peptide/polypeptide/protein(s)	1564	2571	153
inner/outer/membrane/transmembrane	502	751	30
binds/binding	418	1480	21
regulate(s)/regulated/regulator(y)/regulation/regulon	367	897	21
transport/transporter/export/import	295	688	35
enzyme	271	1588	12
signal	267	264	18
initiation	253	409	24
phage/prophage	186	185	64
oxidoreductase/reductase/reduce	139	361	4
transcription/transcriptional	123	382	13
promoter	119	204	4
periplasm/periplasmic	113	356	3
lipoprotein	98	88	5
transferase	92	376	15
resistance	92	242	1
structures/structural	91	206	0
cryptic	76	45	17
lysis	75	360	6
synthesis	74	743	3
biofilm	74	139	2
phosphate	70	819	5
metabolism	61	415	6
codon	58	208	93
sugar	58	98	8
stress	57	186	0
production	57	118	5
aerobic	55	215	0

The counts indicate the number of unique genes for which each phrase appears. Similar words are grouped into sets.

## DISCUSSION

In 1998, a year after the first *E. coli* genome sequence was released, Kenneth Rudd proposed a systematic naming scheme for unannotated open reading frames where each was given a unique name starting with the letter ‘y’ (8). This is a convenient system, but the community did not settle on an official mechanism for assigning new names for these y-genes when functions were established. The tradition has been that new primary names are proposed in the first published report of a newly-identified gene function. This leaves it to peer reviewers to call out duplicate names and other issues. Without a central mechanism for standardized naming, many y-genes have been annotated without receiving new names (173 genes, Figure 2C). And poorly annotated genes have received new names not starting with ‘y’ because their function was partially established, determined based on computational predictions or based on presence in an operon (462 genes, Figure 2C). With the y-ome, we can decouple gene names from assessments of functional annotation and provide a more consistent resource for anyone interested in systematic analysis of unannotated genes.

Future y-ome analysis will be far more precise if the y-ome definition can be formalized in biological knowledge bases. Experimental evidence is already being recorded by evidence ontologies in BioCyc (EcoCyc) (29) and ECO (available in UniProt) (47). However, the implementation details of these ontologies are important for their use in the y-ome. This is best explained with the example of *yihA/engB*, a poorly annotated gene in the y-ome. The ECO evidence ontology describes experimental evidence of GTP binding for *yihA/engB*, and EcoCyc describes the function of the gene as being inferred from a mutant phenotype. The gene is described as having ‘extremely low GTPase activity’ (EcoCyc) and being ‘necessary for normal cell division and for the maintenance of normal septation’ (UniProt). But, the mechanism by which the gene affects cell phenotype is not described in these knowledge bases, so it is included in the y-ome even though evidence ontology information is available. Evidence ontologies are extremely useful, but, to incorporate them into the y-ome, additional mechanistic information is required. A potential solution is to use systems biology models to encode that mechanistic link.

In systems biology, predictive models can be used to link genotype to phenotype. In these models, the definition of functional annotation is that a gene can be mechanistically linked through a network to a measurable phenotypic effect. The contribution of any gene function to cellular phenotype can now be codified computationally for various cellular systems, including metabolism (48), cell signaling (49), gene expression (50) and replication (7). Comparing the 2803 well-annotated genes in *E. coli* to the 1678 genes in the latest genome-scale ME-model (one of the most comprehensive predictive models of *E. coli* to-date) (51), it is clear that the models can grow by over a thousand genes before running up against our lack of knowledge (Figure 2B). To integrate additional biological knowledge, great progress is being made on mechanistic modeling of whole cells (7,52). If the knowledge in whole-cell models can be integrated with evidence ontologies, then it should be possible to greatly improve the precision of the y-ome.

It is worth noting that 75 genes appear in both the ME-model and the y-ome (Figure 2B). Often, genes lacking experimental evidence are included in genome-scale models as part of a ‘gap-filling’ process (53). Usually these genes have some evidence of function (e.g. a known protein family), and the systems biology context indicates that a specific activity is necessary for the cell to function, so the most-likely (low confidence) gene annotation is included in the model. These cases can then be used to drive experimental characterization of low-confidence gene function (25). Thus, integrating predictive models with knowledge bases will provide both clarity on the content of y-ome and insight into the functions of y-ome genes.

The concept of a y-ome can be applied to any genome, and we hope that the y-ome workflow will inspire development of the y-ome of other organisms. For well-characterized model organisms, the workflow presented here can be an initial guide to develop a y-ome. For non-model organisms with few direct experimental studies of gene function, the y-ome will encompass much of the genome. To decrease the size of the y-ome in these organisms, new workflows will be necessary, combining computational genome annotation with systems biology modeling and new high-throughput experimental approaches (26,44,45,54) to establish high-confidence functional annotations across the genome.

## DATA AVAILABILITY

All code and data necessary to reproduce this analysis can be found on GitHub and with a permanent DOI on Zenodo:

- https://github.com/zakandrewking/y-ome
- https://doi.org/10.5281/zenodo.1906044

## Supplementary Material

Supplementary Data
