# Supplementary material for: The y-ome defines the 35% of Escherichia coli genes that lack experimental evidence of function
Source: Nucleic Acids Res. 2019 Jan 30;47(5):2446–54. doi: 10.1093/nar/gkz030 (PMC6412132; doi:10.1093/nar/gkz030)
Supplement: Supplementary Data [file gkz030_supplemental_files.zip › SUPPLEMENTARY DATASETS.pdf]

SUPPLEMENTARY DATASETS

S1 y-ome Genes.tsv

A tab-separated text file containing all genes from this analysis, their locus tags, primary name(s), and whether they are in the "y-ome", "well-annotated", or "excluded" category.

S2 Features.tsv

A tab-separated text file containing all features extracted from the knowledge bases. Each line contains a gene locus tag, the knowledge base name, the gene primary name(s), the feature type for that knowledge base, and the feature itself extracted as text.

S3 No Information.tsv

A tab-separated text file containing a list of 111 genes for which no information could be found in the knowledge bases, with locus tags and EcoCyc primary names.

S4 RNA-seq Conditions.tsv

A tab-separated text file containing descriptions of all RNA-seq conditions and the associated GEO accession numbers.

S5 Annotation Keywords.tsv

A tab-separated text file containing all keywords used in the y-ome workflow. The type indicates whether the keyword was used as an indicator of a y-ome gene ("low") or a well-annotated gene ("high"). The type "high (override)" indicates that this keyword was used to override other annotations in the consensus rules (see Methods). The location column indicates the knowledge base feature where the workflow looks for each keyword. Keywords with type "no information" were used to identify genes for which no information at all is available, and these keywords are provided as Python Regular Expressions which were used to search across all feature columns.

S6 Co-expressed Modules.tsv

A tab-separated text file containing co-expressed gene clusters identified with IterativeWGCNA.
