# Supplementary material for: The y-ome defines the 35% of Escherichia coli genes that lack experimental evidence of function
Source: Nucleic Acids Res. 2019 Jan 30;47(5):2446–54. doi: 10.1093/nar/gkz030 (PMC6412132; doi:10.1093/nar/gkz030)
Supplement: Supplementary Data [file gkz030_supplemental_files.zip › supplementary-figures-.pdf]

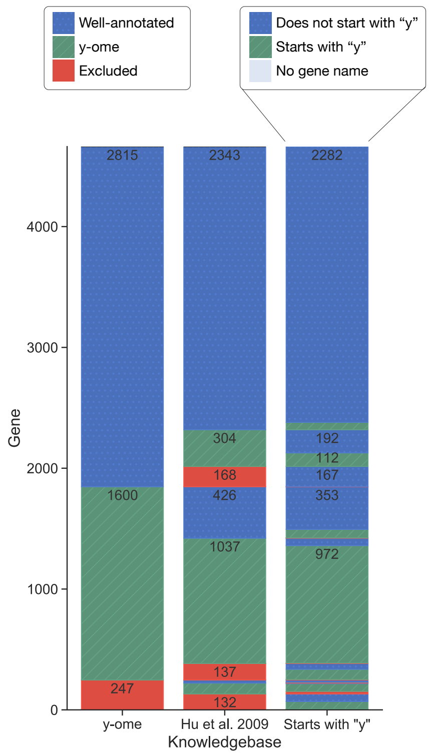

Supplementary Figure S1. A comparison between the y-ome genes and the unannotated gene set used by Hu et al. (2009). The genes whose primary name start with “y” in EcoCyc are also listed.

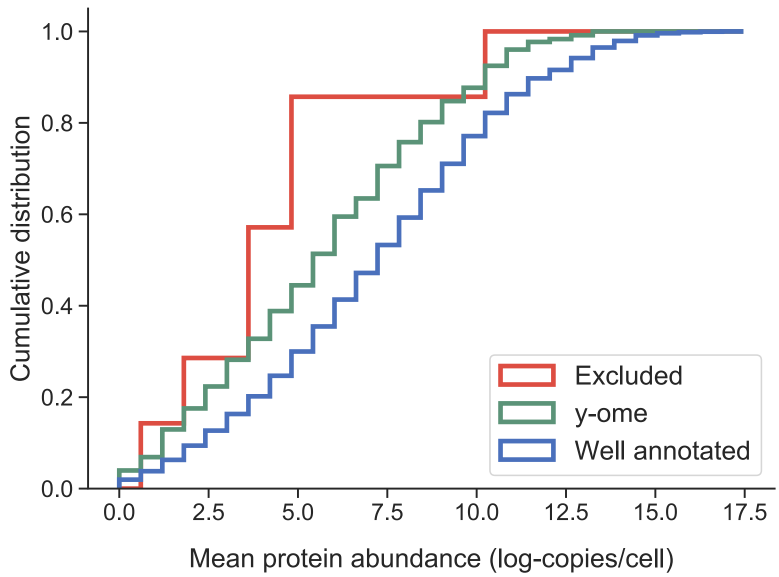

Supplementary Figure S2. Cumulative distribution of mean-log-protein abundances for y-ome (green), well-annotated (blue), and excluded (red) genes across the quantitative proteomics datasets reported by Schmidt et al. (2016). Protein abundances were transformed with log2(x+1) to calculate log-copies/cell, and the means of these values were calculated across conditions.
